# Cortical Oscillatory Signatures Reveal the Prerequisites for Tinnitus Perception: A Comparison of Subjects With Sudden Sensorineural Hearing Loss With and Without Tinnitus

**DOI:** 10.3389/fnins.2020.596647

**Published:** 2020-11-27

**Authors:** Sang-Yeon Lee, Byung Yoon Choi, Ja-Won Koo, Dirk De Ridder, Jae-Jin Song

**Affiliations:** ^1^Department of Otorhinolaryngology-Head and Neck Surgery, Seoul National University Bundang Hospital, Seongnam, South Korea; ^2^Unit of Neurosurgery, Department of Surgical Sciences, Dunedin School of Medicine, University of Otago, Dunedin, New Zealand

**Keywords:** tinnitus, sudden sensorineural hearing loss, bayes, cingulate gyrus, electroencephalography

## Abstract

Just as the human brain works in a Bayesian manner to minimize uncertainty regarding external stimuli, a deafferented brain due to hearing loss attempts to obtain or “fill in” the missing auditory information, resulting in auditory phantom percepts (i.e., tinnitus). Among various types of hearing loss, sudden sensorineural hearing loss (SSNHL) has been extensively reported to be associated with tinnitus. However, the reason that tinnitus develops selectively in some patients with SSNHL remains elusive, which led us to hypothesize that patients with SSNHL with tinnitus (SSNHL-T) and those without tinnitus (SSNHL-NT) may exhibit different cortical activity patterns. In the current study, we compared resting-state quantitative electroencephalography findings between 13 SSNHL-T and 13 SSNHL-NT subjects strictly matched for demographic characteristics and hearing thresholds. By performing whole-brain source localization analysis complemented by functional connectivity analysis, we aimed to determine the as-yet-unidentified cortical oscillatory signatures that may reveal potential prerequisites for the perception of tinnitus in patients with SSNHL. Compared with the SSNHL-NT group, the SSNHL-T group showed significantly higher cortical activity in Bayesian inferential network areas such as the frontopolar cortex, orbitofrontal cortex (OFC), and pregenual anterior cingulate cortex (pgACC) for the beta 3 and gamma frequency bands. This suggests that tinnitus develops in a brain with sudden auditory deafferentation only if the Bayesian inferential network updates the missing auditory information and the pgACC-based top-down gatekeeper system is actively involved. Additionally, significantly increased connectivity between the OFC and precuneus for the gamma frequency band was observed in the SSNHL-T group, further suggesting that tinnitus derived from Bayesian inference may be linked to the default mode network so that tinnitus is regarded as normal. Taken together, our preliminary results suggest a possible mechanism for the selective development of tinnitus in patients with SSNHL. Also, these areas could serve as the potential targets of neuromodulatory approaches to preventing the development or prolonged perception of tinnitus in subjects with SSNHL.

## Introduction

Non-pulsatile tinnitus is a common otologic symptom characterized by conscious auditory perception in the absence of an external stimulus. This is often called a “phantom sound” because there is no corresponding genuine physical source of the sound (Vanneste et al., 2018b; Lee et al., 2019; Han et al., 2020). Although the exact mechanism of tinnitus has yet to be elucidated, peripheral auditory deafferentation has been suggested as the most important factor in increased spontaneous neuronal firing in the central auditory system and cortical maladaptive plasticity between auditory and non-auditory brain regions, leading to the development of tinnitus (Eggermont and Roberts, 2012; Elgoyhen et al., 2015). Hearing loss has been strongly implicated in tinnitus, as demonstrated by a relationship between tinnitus pitch and maximum hearing loss frequency, which suggests that tinnitus is a fill-in phenomenon (Schecklmann et al., 2012). Recently, growing evidence has shown that the brain works in a Bayesian manner to minimize perceptual uncertainty regarding external stimuli. If the brain is deprived of auditory input, it attempts to “fill in” the missing auditory information from auditory memory, leading to the perception of auditory phantoms (i.e., tinnitus) (Friston et al., 2014; Eggermont and Kral, 2016; Lee et al., 2017, 2020a). Specifically, according to the theoretical multiphase compensation model, the brain attempts to overcome missing auditory information input, generating predictions via increasing topographically restricted tones, widening receptive fields, rewiring dendrites and axons, and retrieving auditory memories, resulting in brain reorganization (De Ridder et al., 2014b).

Sudden sensorineural hearing loss (SSNHL), a complex and challenging emergency in the otology field, is typically defined as a sensorineural hearing loss of more than 30 dB across three consecutive frequencies in a pure-tone audiogram occurring within a 72-h period. Importantly, tinnitus was reportedly accompanied by SSNHL in 66–93% of cases (Ding et al., 2018). Similar to ordinary progressive sensorineural hearing loss, the Bayesian brain model may explain how sudden auditory deprivation (i.e., SSNHL) elicits auditory phantom percepts, namely by increasing the need to compensate for prediction errors by upregulating neural firing in specific tonotopic regions and retrieving extant memories from the parahippocampal gyrus (Lee et al., 2017), depending on the amount of hearing loss (Vanneste and De Ridder, 2016). However, why not all patients with SSNHL experience tinnitus remains unexplained. That is, although tinnitus persists in some patients with SSNHL even after treatment, other patients do not experience tinnitus, or tinnitus is perceived temporarily but resolves spontaneously afterward. This, in turn, led us to hypothesize that tinnitus may develop in subjects with SSNHL only if the requisite cortical changes occur secondary to SSNHL.

Zhang et al. demonstrated altered white matter integrity in the auditory neural pathway of patients with SSNHL, which may be associated with the severity of tinnitus (Zhang et al., 2020). Furthermore, a recent study by Cai et al. showed more specific inhibition of neural activity and functional connectivity in patients with SSNHL and tinnitus compared with healthy controls (Cai et al., 2019), shedding further light on the putative association between SSNHL and tinnitus from the perspective of brain activity. However, neural substrates for selective development of tinnitus have thus far not been investigated among patients with SSNHL.

To test this hypothesis, we investigated neural substrates accounting for the development of tinnitus exclusively in patients with SSNHL by comparing resting-state quantitative electroencephalography (rs-qEEG) findings between SSNHL patients with and others without tinnitus (SSNHL-T and SSNHL-NT). Using whole-brain source localization analysis complemented by functional connectivity analysis, we aimed to determine the as-yet-unidentified cortical oscillatory signatures that could reveal the prerequisites for tinnitus development and to discuss the possible mechanism of the selective development of tinnitus in patients with SSNHL. Although this study includes a relatively small number of patients, which may have weakened the clinical implications of the results and statistical power, the results presented herein seem a more significant undertaking than we initially envisioned. Indeed, there is currently no consensus on the neurobiological markers for selective development of tinnitus in patients with SSNHL. Overall, our study stands out in this precision medicine era for incorporating neuroimaging in a tinnitus study to establish a future guide for the treatment of tinnitus in patients with SSNHL that incorporates neuroimaging as the “new normal.”

## Materials and Methods

### Participants

We performed a retrospective review of the medical records of patients with unilateral idiopathic SSNHL who visited the outpatient clinic at Seoul National University Bundang Hospital (SNUBH) between January 2014 and March 2020. For the SSNHL-NT group, we were able to identify only 18 patients who met the criteria for unilateral SSNHL with no complaint of tinnitus. Two of the 18 were excluded due to an insufficient follow-up period (i.e., <2 months). Of the remaining 16 patients, 3 were disqualified due to delayed emergence of tinnitus or significant hearing improvement during the follow-up period of at least 2 months after the onset of SSNHL. Ultimately, 13 patients were enrolled in the SSNHL-NT group. No patients in this group were diagnosed with Meniere's disease, vestibular schwannoma, or psychiatric/neurological disorders.

As outlined in Table 1, 51 SSNHL-T patients whose Tinnitus Handicap Index (THI) score was ≤36 (grades 1 or 2), which was administered to minimize potential bias caused by distress-induced changes in cortical activity, were initially selected from the SNUBH database (1,196 rs-qEEG-available subjects). Subsequently, 13 subjects matched for sex, laterality, and audiogram (i.e., >70 dB HL in the affected ear and <40 dB HL in the unaffected ear) with the SSHL-NT subjects but blinded to rs-qEEG findings were finally enrolled in the SSNHL-T group. None of the subjects in the SSNHL-T group had a history of objective tinnitus or etiologies such as Meniere's disease, head injuries, brain surgery, or neurological disorders. The study was approved by the Institutional Review Board of the Clinical Research Institute at Seoul National University Bundang Hospital and was conducted in accordance with the Declaration of Helsinki (IRB-B-2006-621-105).

**Table 1:** Demographics and clinical characteristics.

	**SSNHL-NT group**	**SSNHL-T group**	***P-*value**
	**(*N* = 13)**	**(*N* = 13)**	
**Age**			
Median	70	62	0.129
Range	30–81	30–74	
**Sex**			
Male	6	6	1.000
Female	7	7	
**Laterality**			
Right	8	8	1.000
Left	5	5	
**Duration (months)^a^**			
Median	16	11	0.696
Range	3–84	3–106	
**Hearing threshold (dB HL)**			
250 Hz	71.15 ± 24.93	60.00 ± 29.86	0.597
500 Hz	80.00 ± 22.82	72.31 ± 24.12	0.037
1 kHz	86.15 ± 14.74	77.31 ± 22.04	0.400
2 kHz	85.00 ± 16.20	83.46 ± 26.09	0.024
3 kHz	89.23 ± 15.53	85.38 ± 27.27	0.588
4 kHz	89.62 ± 15.34	90.77 ± 21.30	0.240
8 kHz	90.77 ± 14.56	94.23 ± 18.53	0.101
**Average hearing threshold (dB HL)^b^**			
Mean (SD)	80.96 ± 20.38	85.19 ± 14.83	0.551
**THI score^b^**			
Mean (SD)	NA	26.46 ± 8.37	NA
Range		8–36	

*SSNHL-NT, sudden sensorineural hearing loss without tinnitus; SSNHL-T, sudden sensorineural hearing loss with tinnitus; SD, standard deviation; HL, hearing loss; THI, tinnitus handicap inventory; NA, not available*.

a *Note that duration refers to the period between SSNHL onset and EEG acquisition*.

b *Note that the mean hearing threshold was calculated using the average of the hearing thresholds at 0.5, 1, 2, and 4 kHz*.

### Audiological and Psychoacoustic Evaluation

The hearing thresholds for seven different octave frequencies (0.25, 0.5, 1, 2, 3, 4, and 8 kHz) were evaluated using pure-tone audiometry in a soundproof booth. The mean hearing threshold was calculated using the average of the hearing thresholds at 0.5, 1, 2, and 4 kHz (Han et al., 2019; Shim et al., 2019; Bae et al., 2020; Huh et al., 2020; Lee et al., 2020b; Song et al., 2020). At each subject's initial visit, we obtained a structured history of the characteristics of tinnitus including its presence, laterality, and psychoacoustic nature (pure-tone or narrow-band noise).

### EEG Recording

We performed qEEG data acquisition and pre-processing procedures according to a previously reported protocol (Kim et al., 2016; Song et al., 2017; Han et al., 2018; Vanneste et al., 2018b; Lee et al., 2019). Prior to EEG recording, we instructed the enrolled patients not to drink alcohol for 24 h and to avoid caffeine on the day of recording to exclude alcohol-induced changes in the EEG signal (Korucuoglu et al., 2016) and caffeine-induced reductions in alpha and beta power (Siepmann and Kirch, 2002). EEGs were recorded with the patient seated upright with the eyes closed for 5 min using a tin-electrode cap (ElectroCap, Eaton, OH, USA), a Mitsar amplifier (EEG-201; Mitsar, St. Petersburg, Russia), and WinEEG software, version 2.84.44 (Mitsar) in a fully lit room insulated from sound and stray electric fields. The EEG data were obtained using WinEEG software (ver. 2.84.44; Mitsar) (available at http://www.mitsar-medical.com). The impedances of all electrodes were maintained below 5 kΩ. Data were obtained at a sampling rate of 1,024 Hz and filtered using a high-pass filter with a cutoff of 0.15 Hz and a low-pass filter with a cutoff of 200 Hz. After initial data acquisition, the raw data were resampled at 128 Hz and band-pass filtered using a fast Fourier transform filter with a Hanning window at 2–44 Hz. After importing the data into Eureka! Software (Sherlin and Congedo, 2005), all episodic artifacts were evaluated manually and removed from the EEG stream. We eliminated additional artifacts using independent component analysis with ICoN software (http://sites.google.com/site/marcocongedo/software/nica) (Koprivova et al., 2011; White et al., 2012). All subjects' vigilance levels, including slowing of alpha rhythm or emergence of sleep spindles, were meticulously monitored. No patients included in this study exhibited any abnormal EEG patterns during the measurements.

### Source Localization Analysis

Standardized low-resolution brain electromagnetic tomography (sLORETA) was employed to estimate the scalp-recorded electrical activity in each of the eight frequency bands (i.e., intracerebral sources). The sLORETA software includes a toolbox for the functional localization of standardized current densities based on electrophysiological and neuroanatomical constraints (Pascual-Marqui, 2002). We identified the cortical sources that generated the activities recorded by the scalp electrodes in each of the following eight frequency bands: delta (2–3.5 Hz), theta (4–7.5 Hz), alpha 1 (8–10 Hz), alpha 2 (10–12 Hz), beta 1 (13–18 Hz), beta 2 (18.5–21 Hz), beta 3 (21.5–30 Hz), and gamma (30.5–44 Hz). sLORETA computes neuronal electrical activity as current density (A/m^2^) without assuming a predefined number of active sources. The sLORETA solution space consists of 6,239 voxels (voxel size: 5 × 5 × 5 mm) and is restricted to the cortical gray matter and hippocampus, as defined by the digitized Montreal Neurological Institute (MNI) 152 template (Fuchs et al., 2002). Scalp electrode coordinates on the MNI brain are derived from the International 5% System (Jurcak et al., 2007). A total of 5,000 random permutations, with correction for multiple testing (i.e., for tests performed for all electrodes and/or voxels and for all time samples and/or different frequencies) were carried out; thus, further correction for multiple comparisons was unnecessary. The locations of significant clusters were confirmed using a LORETA-KEY toolbox, such as the Anatomy toolbox, and the Talairach and Tournoux atlas (Talairach and Tornoux, 1988).

### Functional Connectivity Analysis

As for the functional connectivity analysis, a total of 16 regions of interest, defined by their respective Brodmann areas (BAs) and known to relate to tinnitus according to previously published literature (Vanneste et al., 2018b), were selected as possible nodes. These included the bilateral superior parietal lobule (BA7), the bilateral frontopolar cortices (BA10), the bilateral orbitofrontal cortices (BA11), the bilateral posterior cingulate cortices (BA27), the bilateral pregenual cortices (BA32), the bilateral parahippocampi (BA36), and the bilateral primary auditory cortices (BA41 and BA42).

### Statistical Analyses

Statistical non-parametric mapping (SnPM) was adopted for permutation tests for source localization and functional connectivity. To identify between-group differences in resting-state cortical oscillatory activities, sLORETA built-in voxel-wise randomization tests (5,000 permutations) were used to perform nonparametric statistical analyses of functional images with a threshold *P* < 0.05. We also employed a between-groups *t*-statistic with a threshold of *P* < 0.05. Correction for multiple comparisons in SnPM using random permutations has been shown to yield similar results to those obtained from a statistical parametric mapping approach using a general linear model with multiple-comparison corrections (Nichols and Holmes, 2002). For lagged linear connectivity differences, we assessed between-group differences for each contrast using a paired *t*-test with a threshold of *P* < 0.05. We also corrected for multiple comparisons using sLORETA's built-in voxel-wise randomization tests for all of the voxels included in the 16 regions of interests for connectivity analysis (5,000 permutations). Although the between-groups t-statistic was used for source localization and the paired *t*-test was used for connectivity analysis, these are nonparametric analyses based on 5,000 permutations. All analyses were done and illustrated using the R statistical package (version 3.3.2, R Foundation for Statistical Computing, Vienna, Austria). All statistical tests were two-tailed, and *P* < 0.05 was considered significant.

## Results

### Demographics and Clinical Characteristics: SSNHL-T vs. SSNHL-NT

**Figure 1:**
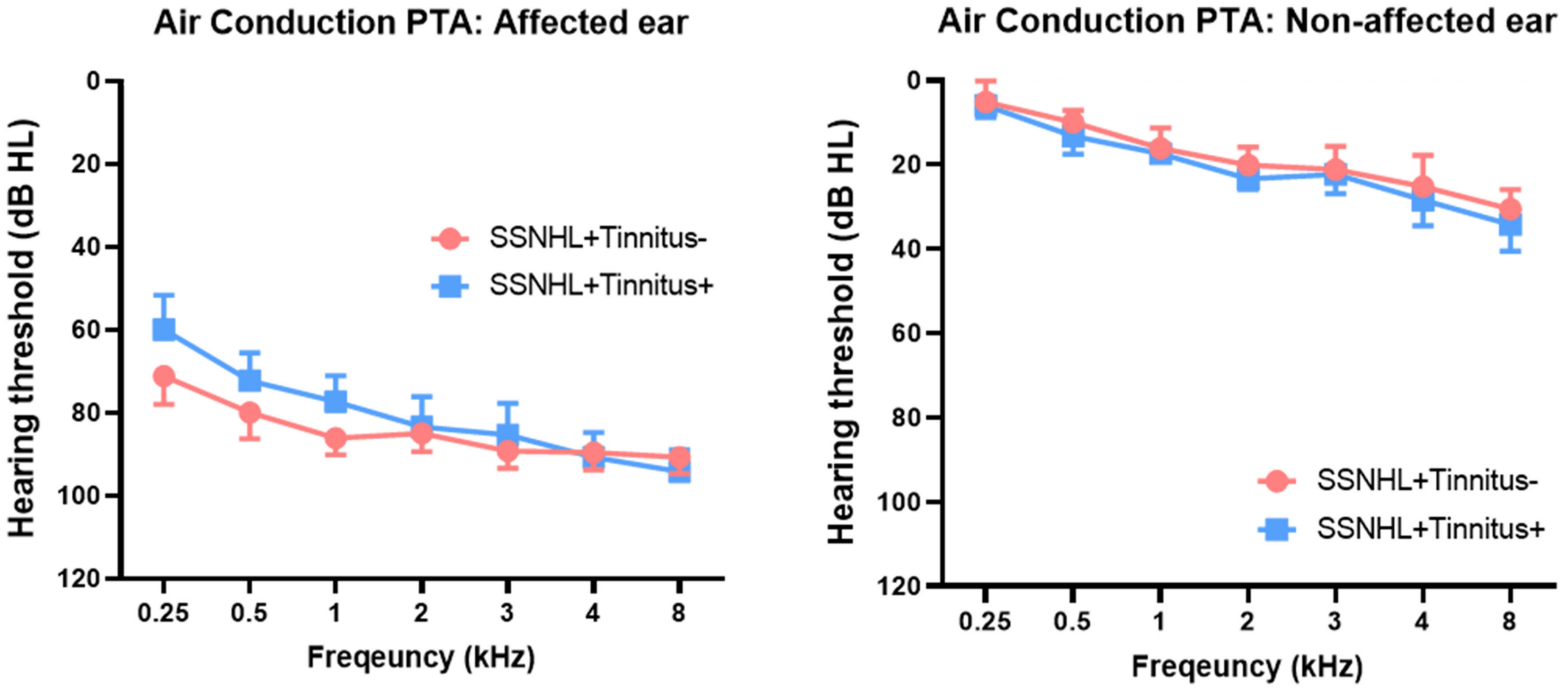
Comparison of hearing thresholds across all frequencies between patients with sudden sensorineural hearing loss with and without tinnitus (SSNHL-T and SSNHL-NT, respectively). Air conduction pure-tone audiometry (PTA) revealed nearly matched hearing thresholds across all frequencies between the two groups in both the affected and the non-affected ear.

The demographic and clinical characteristics of the two groups are summarized in Figure 1. The laterality of the hearing loss and sex distribution were matched between the SSNHL-NT and SSNHL-T groups. No significant differences in age at onset of SSNHL or duration of hearing loss (from the onset of SSNHL to the timepoint at rs-qEEG measurement) were observed between the two groups. Furthermore, hearing thresholds across all frequencies for the affected- and non-affected ears did not differ between the two groups. The median THI score of the SSNHL-T group was 12 (range, 4–36).

### Source-Localization Analysis: SSNHL-T vs. SSNHL-NT

**Figure 2:**
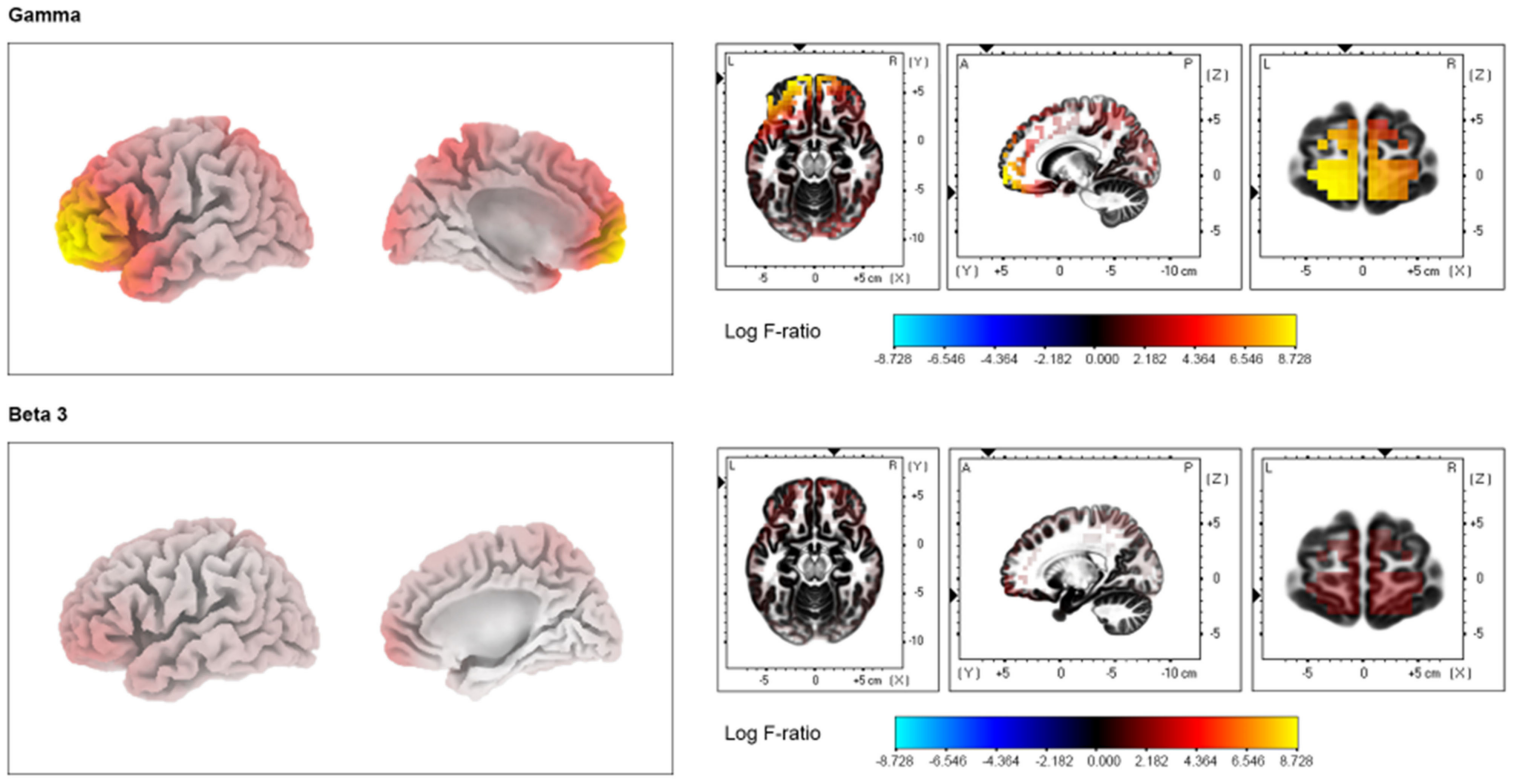
Source-localized cortical power comparison in sudden sensorineural hearing loss with and without tinnitus (SSNHL-T and SSNHL-NT, respectively) groups using resting-state quantitative electroencephalography data. The SSNHL-T group showed increased activity in the frontopolar cortex, orbitofrontal cortex, and pregenual anterior cingulate cortex for the gamma and beta 3 frequency bands compared with the SSNHL-NT group.

Compared with the SSNHL-NT group, the SSNHL-T group showed significantly increased cortical activity in the frontopolar cortex (FPC, BA10), the orbitofrontal cortex (OFC, BA11), and the pregenual anterior cingulate cortex (pgACC, BA32) for the beta 3 and gamma frequency bands (*P* < 0.05) (Figure 2). No significant effects were observed for the delta, theta, alpha 1, alpha 3, beta 1, and beta 2 frequency bands.

### Connectivity Analyses: SSNHL-T vs. SSNHL-T

**Figure 3:**
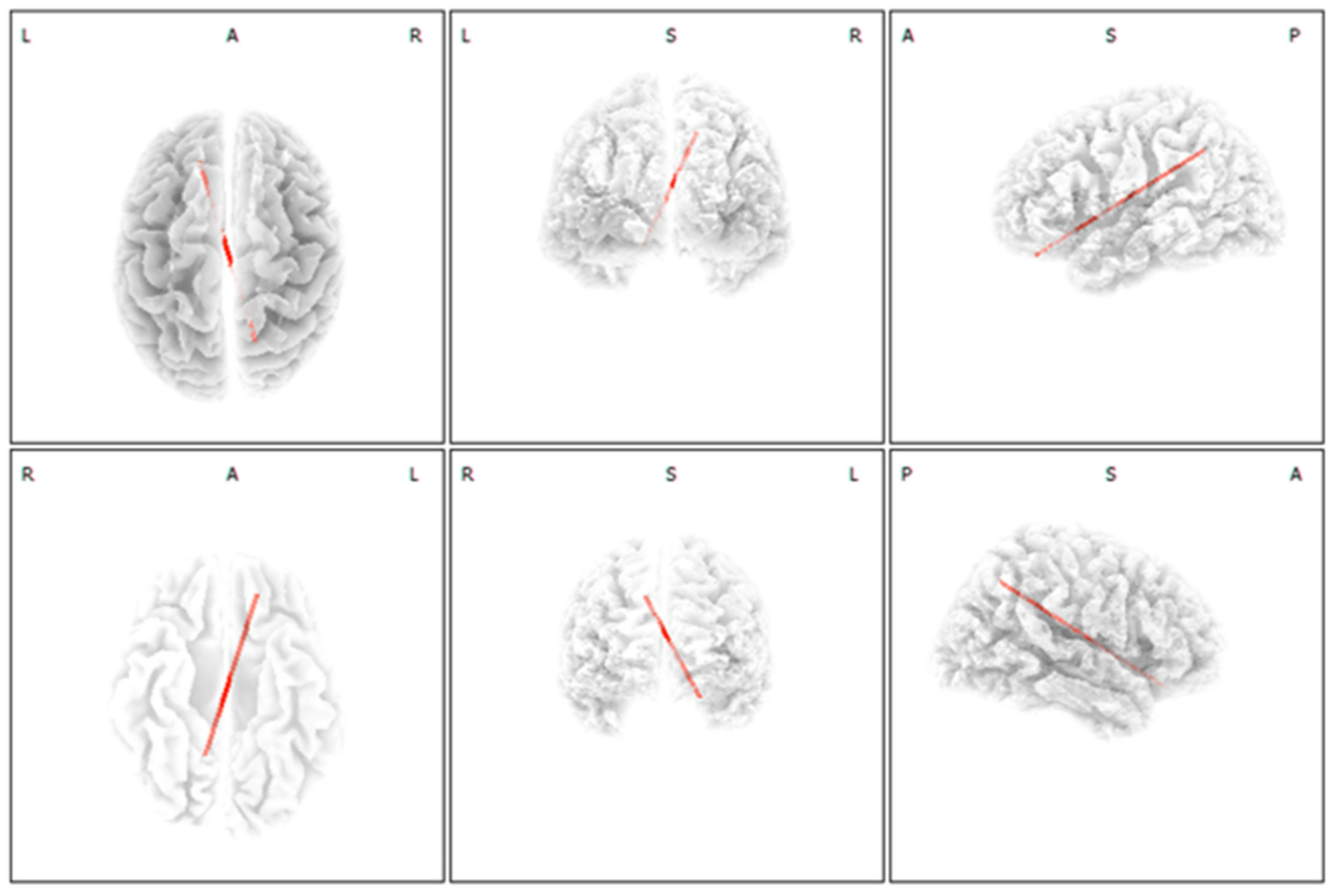
Functional connectivity analysis with regard to selective development of tinnitus in subjects with sudden sensorineural hearing loss (SSNHL). Increased functional connectivity between the left orbitofrontal cortex and the right precuneus for the gamma frequency band was significant in the SSNHL with tinnitus (SSNHL-T) compared with the SSNHL without tinnitus (SSNHL-NT) group.

Compared with the SSNHL-NT group, the SSNHL-T group showed significantly increased functional connectivity between the left OFC and the right precuneus (BA7) for the gamma frequency band (*P* < 0.05) (Figure 3). For the other seven frequency bands, there were no significant between-group differences in functional connectivity among ROIs.

## Discussion

This is the first study to explore cortical activity and connectivity differences between SSNHL subjects with and those without tinnitus and to attempt to reveal the cortical oscillatory signatures for selective development of tinnitus among patients with SSNHL. In this study, the SSNHL-T group had abnormally increased activity in the FPC, OFC, and pgACC for the beta 3 and gamma frequency bands compared with the SSNHL-NT group. These findings suggest that auditory phantom percepts may develop when the brain experiences sudden decreased peripheral auditory input as the Bayesian inferential network updates the missing auditory information with the involvement of the pgACC-based top-down gatekeeper system. Furthermore, the lagged linear connectivity between the left OFC and the right precuneus was significantly increased for the gamma frequency band in the SSNHL-T group compared with the SSNHL-NT group, indicating that tinnitus deriving from Bayesian updating seems to involve the default mode network (DMN); thus, tinnitus seemed to be perceived as normal by the SSNHL-T group.

### The Bayesian Inferential Network Updates Missing Auditory Information via Bottom-Up Deafferentation

The Bayesian brain model, an extension of a predictive brain model, has been suggested as an explanation for the development of tinnitus. According to this model, tinnitus is a response to peripheral auditory deafferentation that aims to reduce perceptual uncertainty (Morcom and Friston, 2012; De Ridder et al., 2014b). In other words, deafferentation-induced auditory phantom percepts, namely tinnitus, are preceded by peripheral auditory input-based memory, and tinnitus develops when prediction error occurs due to peripheral hearing loss (De Ridder et al., 2014a; Lee et al., 2017). In the same context, we have recently reported that approximately 70% of patients with unilateral SSNHL experience ipsilesional tinnitus (Lee et al., 2017), indicating that missing auditory information (i.e., prediction error) may stimulate neural circuit interactions between lower-order (peripheral auditory input) and higher-order (prediction-driving process of auditory perception) auditory systems to reduce uncertainty in a bottom-up fashion due to sudden hearing deterioration. In this regard, significantly increased source-localized activity in the OFC and FPC in the SSNHL-T group in the current study may reflect the role of active Bayesian inferential prefrontal cortical processes (Donoso et al., 2014) in tinnitus generation in the context of a sudden decrease in peripheral auditory input. The prefrontal cortices are considered to employ probabilistic inferential processes (i.e., Bayesian inferences), enabling optimizing behavioral adaptations in uncertain situations based on available information (Koechlin, 2016; Parr et al., 2018). In particular, polar to lateral prefrontal cortices such as the OFC and FPC are involved in making probabilistic inferences and exploring new strategies formed from long-term memory in uncertain environments (Donoso et al., 2014). Therefore, increased source-localized activity in the prefrontal cortices (i.e., OFC and FPC) in the SSNHL-T group may reflect the Bayesian inferential processes of updating sensory prediction and thereby adopting new strategies (phantom auditory perception) based on stored auditory memory in the context of suddenly decreased peripheral auditory input. Of note, we have recently revealed significantly increased information inflow in cortical areas associated with Bayesian inference in progressive sensorineural hearing loss patients with tinnitus as compared to those without tinnitus (unpublished data), in accordance with the current findings.

The OFC has also been suggested as responsible for the emotional processing of sounds (Blood et al., 1999), and is connected to other limbic areas involved in emotion processing (Beauregard, 2007; Vanneste and De Ridder, 2012). In an integrative model of tinnitus (De Ridder et al., 2014c), once the aberrant activity that causes tinnitus percepts is deemed salient, the autonomic nervous system, the limbic system, and their interaction could be further involved in distributing tinnitus-related distress signals across the brain. Indeed, the OFC has been reported to play a pivotal role in the top-down modulation of autonomic and peripheral physiological responses accompanying emotional experiences (Ohira et al., 2006), supporting neural activity in the OFC might link to biopsychosocial processes of disease (Hänsel and von Känel, 2008). Furthermore, neural activity in the OFC extending to the FPC in beta 1 and beta 2 has shown to differ between sex during emotional processing and emotional regulation (Vanneste et al., 2012). Additionally, tinnitus perception and tinnitus-related distress are closely associated with these brain areas (Schlee et al., 2009), and correlated with the audiological handicap associated with unilateral SSNHL.

Additionally, tinnitus loudness and distress are correlated with the audiological handicap associated with unilateral SSNHL (Chiossoine-Kerdel et al., 2000). Although we attempted to minimize distress-related cortical changes by recruiting SSNHL-T subjects with only mild distress, distress cannot be completely eliminated in tinnitus. In this regard, the activity changes in the FPC and OFC may also reflect the emotional weight attached to aberrant auditory perception (i.e., tinnitus) in patients with SSNHL.

### A Top-Down Gatekeeper System Is Activated to Cancel Internally Generated Auditory Phantoms

Recent studies have suggested that auditory phantom percepts can be associated with bottom-up (ascending) deafferentation as well as with a dysfunctional top-down (descending) noise-canceling mechanism (De Ridder et al., 2014b; Song et al., 2015; Vanneste et al., 2019). This top-down mechanism is a putative central gatekeeper that functions as an “auditory gate,” evaluating the relevance and affective meaning of sensory stimuli and modulating information transmission via descending inhibitory pathways to the thalamic reticular nucleus (Hullfish et al., 2019; Vanneste et al., 2019). In previous pain studies, the degree of improvement after spinal cord stimulation depended on activation of the pgACC (Moens et al., 2012), which is a part of the descending pain inhibitory pathway (Fields, 2004; Kong et al., 2010), the somatosensory analog of the noise canceling system. Additionally, Vanneste et al. demonstrated that altered neural activity of the pgACC likely increases tinnitus loudness in patients who are Met carriers (i.e., COMT Val^158^Met polymorphism), probably due to reduced canceling-out of irrelevant auditory input. Furthermore, increased activity in the parahippocampus and the pgACC for the theta and gamma frequency bands, as well as decreased activity in the auditory cortex, is found exclusively in tinnitus patients with hearing loss compared with those who have hearing loss but without tinnitus (Vanneste et al., 2018a). While the activation of a top-down noise-canceling mechanism works predominantly in the alpha frequency band during the resting state, dysfunctional noise canceling resulting in tinnitus is hypothesized to be linked to the theta and gamma frequency bands (Vanneste et al., 2019). These findings are consistent with our data showing increased source-localized activity in the pgACC for the gamma frequency band in the SSNHL-T group. That is, the pgACC, which normally functions as a central gatekeeper, is activated to abate behaviorally irrelevant phantom auditory signals that stem from Bayesian updating via bottom-up deafferentation.

Overall, our data suggest that auditory phantom percepts may develop in a brain with suddenly decreased peripheral auditory input when the Bayesian inferential network actively updates the missing auditory information. Furthermore, as an attempt to minimize this auditory phantom, the pgACC-based top-down gatekeeper system may be activated in brains with sudden auditory deafferentation.

### Tinnitus Percepts May Be Considered the Norm When Bayesian Updating-Based Tinnitus Is Actively Linked to the Default Mode Network

As shown in Figure 3, a significant increase in connectivity between the OFC (BA11) and the precuneus (BA7) was observed in the SSNHL-T group compared with the SSNHL-NT group. The posterior cingulate cortex and precuneus are considered critical nodes of the brain's DMN, a specific group of brain regions activated when people are occupied with an internally focused task (i.e., the task-negative mode) (Vanneste and De Ridder, 2012). Therefore, our data may indicate that patients with SSNHL perceive tinnitus when Bayesian updating-based tinnitus is actively linked to the DMN. The DMN may regard the salient but irrelevant auditory information (i.e., tinnitus) arising from the Bayesian updating as normal, ultimately leading to continuous tinnitus perception. We have recently shown that localized activation of brain areas involved in the DMN may act as a negative predictor of improvement in tinnitus after partial auditory reafferentation by the use of hearing aids or cochlear implants, as tinnitus perception may already seem normal due to activation of DMN-related brain areas (Song et al., 2013; Han et al., 2020). Collectively, these findings reinforce the existing notion that the brain regions involved in generating tinnitus may become integrated into the DMN in patients with tinnitus (De Ridder et al., 2011; Vanneste and De Ridder, 2012). Based on the literature as well as the current findings, our results justify the evaluation of localized activity and functional connectivity using functional neuroimaging in patients with SSNHL. The rationale behind such an effort lies in the expectation that altered brain activity and connectivity, including that of the DMN, may predict the prognosis with regard to the chronification of tinnitus or treatment responses in subjects with tinnitus.

### Limitations and Future Perspectives

Taken together, the results of the present study merit special attention in that they are grossly in line with the recently proposed Bayesian brain model for the generation of tinnitus and offer a key to unraveling the conundrum of the selective development of tinnitus in patients with SSNHL. Our study also raises an important issue that may stimulate further research incorporating customized neuromodulation approaches based on the status of neural substrates responsible for the perception of tinnitus in patients with SSNHL.

Nevertheless, there are some limitations that should be addressed in future studies. First, the results presented here are limited by the relatively small number of subjects in both groups, mainly due to the difficulty of recruiting SSNHL patients without tinnitus. Future follow-up studies in a larger number of subjects should be performed to replicate the current results. Additionally, the current study was designed as a cross-sectional evaluation, which, along with the retrospective study design, may weaken the clinical implications of our results. These limitations require future prospective and longitudinal follow-up studies to determine the origination of these differences of cortical activity and connectivity between SSNHL subjects with and those without tinnitus. Particularly, recruiting patients showing immediate tinnitus following sudden auditory deprivation but improved thereafter, as negative plasticity compensates for itself, would be important to elicit more significant findings. Second, confounding related to distress-induced cortical activity changes was minimized by including SSNHL subjects with tinnitus who had low THI scores; however, such confounding was not completely eliminated because tinnitus with no distress is almost nonexistent. A future prospective study including SSNHL with “very minimally” distressing tinnitus should be conducted to confirm the reproducibility of the current findings. Third, this study did not consider the possibility of combined hyperacusis in the SSNHL-T group. A recent study using rs-qEEG showed that increased “circuit-breaker” activity was associated with hyperacusis-related neural substrates (Han et al., 2018), which suggests that cortical activity may be biased if tinnitus subjects with combined hyperacusis are included. Future studies recruiting a SSNHL-T group without combined hyperacusis should be performed to address this limitation.

## Conclusion

Our preliminary study explored cortical activity and connectivity differences between SSNHL subjects with and without tinnitus, shedding light on the cortical oscillatory signatures for selective development of tinnitus among patients with SSNHL. These areas could serve as potential targets of neuromodulatory approaches to prevent the development or prolonged perception of tinnitus in subjects with SSNHL.

## Data Availability Statement

The original contributions presented in the study are included in the article/supplementary materials, further inquiries can be directed to the corresponding author/s.

## Ethics Statement

The study was approved by the Institutional Review Board of the Clinical Research Institute at Seoul National University Bundang Hospital, and was conducted in accordance with the Declaration of Helsinki (IRB-B-2006-621-105). The patients/participants provided their written informed consent to participate in this study.

## Author Contributions

S-YL and J-JS led the analysis and interpretation of the results and drafted the first manuscript. BC, J-WK, and DD conceived the investigation and revised the manuscript for important intellectual content. All authors contributed to all aspects of the investigation, including methodological design, data collection and analysis, interpretation of the results, revision of the manuscript for important intellectual content, and approved the final version of the manuscript and agree to be accountable for all aspects of the work.

## Conflict of Interest

The authors declare that the research was conducted in the absence of any commercial or financial relationships that could be construed as a potential conflict of interest.

## References

[B1] Bae E. B. Lee J. H. Song J. J. (2020). Single-session of combined tDCS-TMS may increase therapeutic effects in subjects with tinnitus. Front. Neurol. 11:160. 10.3389/fneur.2020.00160 32292383 PMC7118567

[B2] Beauregard M. (2007). Mind does really matter: evidence from neuroimaging studies of emotional self-regulation, psychotherapy, and placebo effect. Prog. Neurobiol. 81, 218–236. 10.1016/j.pneurobio.2007.01.005 17349730

[B3] Blood A. J. Zatorre R. J. Bermudez P. Evans A. C. (1999). Emotional responses to pleasant and unpleasant music correlate with activity in paralimbic brain regions. Nat. Neurosci. 2, 382–387. 10.1038/7299 10204547

[B4] Cai Y. Li J. Chen Y. Chen W. Dang C. Zhao F.. (2019). Inhibition of brain area and functional connectivity in idiopathic sudden sensorineural hearing loss with tinnitus based on resting-state EEG. Front. Neurosci. 13:851. 10.3389/fnins.2019.00851 31474821 PMC6702325

[B5] Chiossoine-Kerdel J. A. Baguley D. M. Stoddart R. L. Moffat D. A. (2000). An investigation of the audiologic handicap associated with unilateral sudden sensorineural hearing loss. Otol. Neurotol. 21, 645–651. 10993452

[B6] De Ridder D. Elgoyhen A. B. Romo R. Langguth B. (2011). Phantom percepts: tinnitus and pain as persisting aversive memory networks. Proc. Natl. Acad. Sci. 108, 8075–8080. 10.1073/pnas.1018466108 21502503 PMC3100980

[B7] De Ridder D. Joos K. Vanneste S. (2014a). The enigma of the tinnitus-free dream state in a Bayesian world. Neural Plast. 2014:612147. 10.1155/2014/612147 25097788 PMC4109081

[B8] De Ridder D. Vanneste S. Freeman W. (2014b). The Bayesian brain: phantom percepts resolve sensory uncertainty. Neurosci. Biobehav. Rev. 44, 4–15. 10.1016/j.neubiorev.2012.04.001 22516669

[B9] De Ridder D. Vanneste S. Weisz N. Londero A. Schlee W. Elgoyhen A. B.. (2014c). An integrative model of auditory phantom perception: tinnitus as a unified percept of interacting separable subnetworks. Neurosci. Biobehav. Rev. 44, 16–32. 10.1016/j.neubiorev.2013.03.021 23597755

[B10] Ding X. Zhang X. Huang Z. Feng X. (2018). The characteristic and short-term prognosis of tinnitus associated with sudden sensorineural hearing loss. Neural Plast. 2018:6059697. 10.1155/2018/6059697 29861716 PMC5971248

[B11] Donoso M. Collins A. G. Koechlin E. (2014). Human cognition. Foundations of human reasoning in the prefrontal cortex. Science 344, 1481–1486. 10.1126/science.1252254 24876345

[B12] Eggermont J. J. Kral A. (2016). Somatic memory and gain increase as preconditions for tinnitus: insights from congenital deafness. Hear. Res. 333, 37–48. 10.1016/j.heares.2015.12.018 26719143

[B13] Eggermont J. J. Roberts L. E. (2012). The neuroscience of tinnitus: understanding abnormal and normal auditory perception. Front. Syst. Neurosci. 6:53. 10.3389/fnsys.2012.00053 22798948 PMC3394370

[B14] Elgoyhen A. B. Langguth B. De Ridder D. Vanneste S. (2015). Tinnitus: perspectives from human neuroimaging. Nat. Rev. Neurosci. 16, 632–642. 10.1038/nrn4003 26373470

[B15] Fields H. (2004). State-dependent opioid control of pain. Nat. Rev. Neurosci. 5, 565–575. 10.1038/nrn1431 15208698

[B16] Friston K. J. Stephan K. E. Montague R. Dolan R. J. (2014). Computational psychiatry: the brain as a phantastic organ. Lancet Psychiatry 1, 148–158. 10.1016/S2215-0366(14)70275-5 26360579

[B17] Fuchs M. Kastner J. Wagner M. Hawes S. Ebersole J. S. (2002). A standardized boundary element method volume conductor model. Clin. Neurophysiol. 113, 702–712. 10.1016/S1388-2457(02)00030-5 11976050

[B18] Han J. J. Jang J. H. Ridder D. D. Vanneste S. Koo J.-W. Song J.-J. (2018). Increased parietal circuit-breaker activity in delta frequency band and abnormal delta/theta band connectivity in salience network in hyperacusis subjects. PLoS ONE 13:e0191858. 10.1371/journal.pone.0191858 29370266 PMC5785008

[B19] Han J. J. Ridder D. D. Vanneste S. Chen Y.-C. Koo J.-W. Song J.-J. (2020). Pre-treatment ongoing cortical oscillatory activity predicts improvement of tinnitus after partial peripheral reafferentation with hearing aids. Front. Neurosci. 14:410. 10.3389/fnins.2020.00410 32457569 PMC7221249

[B20] Han S. A. Choe G. Kim Y. Koo J. W. Choi B. Y. Song J. J. (2019). Beware of a fragile footplate: lessons from ossiculoplasty in patients with ossicular anomalies related to second pharyngeal arch defects. J. Clin. Med. 8:2130. 10.3390/jcm8122130 31816982 PMC6947221

[B21] Hänsel A. von Känel R. (2008). The ventro-medial prefrontal cortex: a major link between the autonomic nervous system, regulation of emotion, and stress reactivity? Biopsychosoc. Med. 2:21. 10.1186/1751-0759-2-21 18986513 PMC2590602

[B22] Huh G. Bae Y. J. Woo H. J. Park J. H. Koo J. W. Song J. J. (2020). Vestibulocochlear symptoms caused by vertebrobasilar dolichoectasia. Clin. Exp. Otorhinolaryngol. 13, 123–132. 10.21053/ceo.2019.00780 31522490 PMC7248613

[B23] Hullfish J. Abenes I. Yoo H. B. De Ridder D. Vanneste S. (2019). Frontostriatal network dysfunction as a domain-general mechanism underlying phantom perception. Hum. Brain Mapp. 40, 2241–2251. 10.1002/hbm.24521 30648324 PMC6865744

[B24] Jurcak V. Tsuzuki D. Dan I. (2007). 10/20, 10/10, and 10/5 systems revisited: their validity as relative head-surface-based positioning systems. Neuroimage 34, 1600–1611. 10.1016/j.neuroimage.2006.09.024 17207640

[B25] Kim S. H. Jang J. H. Lee S. Y. Han J. J. Koo J. W. Vanneste S.. (2016). Neural substrates predicting short-term improvement of tinnitus loudness and distress after modified tinnitus retraining therapy. Sci. Rep. 6:29140. 10.1038/srep29140 27381994 PMC4933976

[B26] Koechlin E. (2016). Prefrontal executive function and adaptive behavior in complex environments. Curr. Opin. Neurobiol. 37, 1–6. 10.1016/j.conb.2015.11.004 26687618

[B27] Kong J. Loggia M. L. Zyloney C. Tu P. Laviolette P. Gollub R. L. (2010). Exploring the brain in pain: activations, deactivations and their relation. Pain 148, 257–267. 10.1016/j.pain.2009.11.008 20005043 PMC2815185

[B28] Koprivova J. Congedo M. Horacek J. Prasko J. Raszka M. Brunovsky M.. (2011). EEG source analysis in obsessive-compulsive disorder. Clin. Neurophysiol. 122, 1735–1743. 10.1016/j.clinph.2011.01.051 21354363

[B29] Korucuoglu O. Gladwin T. E. Wiers R. W. (2016). The effect of acute alcohol on motor-related EEG asymmetries during preparation of approach or avoid alcohol responses. Biol. Psychol. 114, 81–92. 10.1016/j.biopsycho.2015.12.012 26762699

[B30] Lee J. M. Kim Y. Ji J.-Y. Koo J.-W. Song J.-J. (2020a). “Auditory experience, for a certain duration, is a prerequisite for tinnitus: lessons from subjects with unilateral tinnitus in the better-hearing ear,” in Progress in Brain Research, eds Schlee W. Langguth B. Kleinjung T. Vanneste S. De Ridder D. (Amsterdam: Elsevier). 10.1016/bs.pbr.2020.07.013 33637219

[B31] Lee S. Y. Kim M. K. Bae Y. J. An G. S. Lee K. Choi B. Y.. (2020b). Longitudinal analysis of surgical outcome in subjects with pulsatile tinnitus originating from the sigmoid sinus. Sci. Rep. 10:18194. 10.1038/s41598-020-75348-3 33097817 PMC7584625

[B32] Lee S. Y. Nam D. W. Koo J. W. De Ridder D. Vanneste S. Song J. J. (2017). No auditory experience, no tinnitus: lessons from subjects with congenital- and acquired single-sided deafness. Hear. Res. 354, 9–15. 10.1016/j.heares.2017.08.002 28826043

[B33] Lee S. Y. Rhee J. Shim Y. J. Kim Y. Koo J. W. De Ridder D.. (2019). Changes in the resting-state cortical oscillatory activity 6 months after modified tinnitus retraining therapy. Front. Neurosci. 13:1123. 10.3389/fnins.2019.01123 31680845 PMC6813998

[B34] Moens M. Sunaert S. Marien P. Brouns R. De Smedt A. Droogmans S.. (2012). Spinal cord stimulation modulates cerebral function: an fMRI study. Neuroradiology 54, 1399–1407. 10.1007/s00234-012-1087-8 22941431

[B35] Morcom A. M. Friston K. J. (2012). Decoding episodic memory in ageing: a Bayesian analysis of activity patterns predicting memory. Neuroimage 59, 1772–1782. 10.1016/j.neuroimage.2011.08.071 21907810 PMC3236995

[B36] Nichols T. E. Holmes A. P. (2002). Nonparametric permutation tests for functional neuroimaging: a primer with examples. Hum. Brain Mapp. 15, 1–25. 10.1002/hbm.1058 11747097 PMC6871862

[B37] Ohira H. Nomura M. Ichikawa N. Isowa T. Iidaka T. Sato A.. (2006). Association of neural and physiological responses during voluntary emotion suppression. Neuroimage 29, 721–733. 10.1016/j.neuroimage.2005.08.047 16249100

[B38] Parr T. Rees G. Friston K. J. (2018). Computational neuropsychology and Bayesian inference. Front. Hum. Neurosci. 12:61. 10.3389/fnhum.2018.00061 29527157 PMC5829460

[B39] Pascual-Marqui R. D. (2002). Standardized low-resolution brain electromagnetic tomography (sLORETA): technical details. Methods Find. Exp. Clin. Pharmacol. 24 (Suppl. D), 5–12. 12575463

[B40] Schecklmann M. Vielsmeier V. Steffens T. Landgrebe M. Langguth B. Kleinjung T. (2012). Relationship between audiometric slope and tinnitus pitch in tinnitus patients: insights into the mechanisms of tinnitus generation. PLoS ONE 7:e34878. 10.1371/journal.pone.0034878 22529949 PMC3329543

[B41] Schlee W. Hartmann T. Langguth B. Weisz N. (2009). Abnormal resting-state cortical coupling in chronic tinnitus. BMC Neurosci. 10:11 10.1186/1471-2202-10-11 19228390 PMC2649130

[B42] Sherlin L. Congedo M. (2005). Obsessive-compulsive dimension localized using low-resolution brain electromagnetic tomography (LORETA). Neurosci. Lett. 387, 72–74. 10.1016/j.neulet.2005.06.069 16061322

[B43] Shim Y. J. Bae Y. J. An G. S. Lee K. Kim Y. Lee S. Y.. (2019). Involvement of the internal auditory canal in subjects with cochlear otosclerosis: a less acknowledged third window that affects surgical outcome. Otol. Neurotol. 40, e186–e190. 10.1097/MAO.0000000000002144 30741893

[B44] Siepmann M. Kirch W. (2002). Effects of caffeine on topographic quantitative EEG. Neuropsychobiology 45, 161–166. 10.1159/000054958 11979068

[B45] Song J.-J. Punte A. K. De Ridder D. Vanneste S. Van de Heyning P. (2013). Neural substrates predicting improvement of tinnitus after cochlear implantation in patients with single-sided deafness. Hear. Res. 299, 1–9. 10.1016/j.heares.2013.02.001 23415916

[B46] Song J. J. Kim K. Sunwoo W. Mertens G. Van de Heyning P. De Ridder D.. (2017). A quantitative electroencephalography study on cochlear implant-induced cortical changes in single-sided deafness with tinnitus. Front. Hum. Neurosci. 11:210. 10.3389/fnhum.2017.00210 28572760 PMC5435818

[B47] Song J. J. Ku E. J. Kim S. Kim E. Choi Y. S. Jung H. J. (2020). Increased risk of psychopathological abnormalities in subjects with unilateral hearing loss: a cross-sectional study. Clin. Exp. Otorhinolaryngol. 10.21053/ceo.2020.00283. [Epub ahead of print]. 32734740 PMC7904432

[B48] Song J. J. Vanneste S. De Ridder D. (2015). Dysfunctional noise cancelling of the rostral anterior cingulate cortex in tinnitus patients. PLoS ONE 10:e0123538. 10.1371/journal.pone.0123538 25875099 PMC4395158

[B49] Talairach J. Tornoux P. (1988). Co-Planar Stereotaxic Atlas of the Human Brain: 3-Dimensional Proportional System: An Approach to Cerebral Imaging. Stuttgart: Georg Thieme.

[B50] Vanneste S. Alsalman O. De Ridder D. (2018a). COMT and the neurogenetic architecture of hearing loss induced tinnitus. Hear. Res. 365, 1–15. 10.1016/j.heares.2018.05.020 29883832

[B51] Vanneste S. Alsalman O. De Ridder D. (2019). Top-down and bottom-up regulated auditory phantom perception. J. Neurosci. 39, 364–378. 10.1523/JNEUROSCI.0966-18.2018 30389837 PMC6360282

[B52] Vanneste S. De Ridder D. (2012). The auditory and non-auditory brain areas involved in tinnitus. An emergent property of multiple parallel overlapping subnetworks. Front. Syst. Neurosci. 6:31. 10.3389/fnsys.2012.00031 22586375 PMC3347475

[B53] Vanneste S. De Ridder D. (2016). Deafferentation-based pathophysiological differences in phantom sound: tinnitus with and without hearing loss. Neuroimage 129, 80–94. 10.1016/j.neuroimage.2015.12.002 26708013

[B54] Vanneste S. Joos K. De Ridder D. (2012). Prefrontal cortex based sex differences in tinnitus perception: same tinnitus intensity, same tinnitus distress, different mood. PLoS ONE 7:e31182. 10.1371/journal.pone.0031182 22348053 PMC3277500

[B55] Vanneste S. Song J. J. De Ridder D. (2018b). Thalamocortical dysrhythmia detected by machine learning. Nat. Commun. 9:1103. 10.1038/s41467-018-02820-0 29549239 PMC5856824

[B56] White D. J. Congedo M. Ciorciari J. Silberstein R. B. (2012). Brain oscillatory activity during spatial navigation: theta and gamma activity link medial temporal and parietal regions. J. Cogn. Neurosci. 24, 686–697. 10.1162/jocn_a_00098 21812639

[B57] Zhang Z. Jia X. Guan X. Zhang Y. Lyu Y. Yang J.. (2020). White matter abnormalities of auditory neural pathway in sudden sensorineural hearing loss using diffusion spectrum imaging: different findings from tinnitus. Front. Neurosci. 14:200. 10.3389/fnins.2020.00200 32269506 PMC7109467

